# The tibetan medicine Zuozhu-Daxi can prevent *Helicobacter pylori* induced-gastric mucosa inflammation by inhibiting lipid metabolism

**DOI:** 10.1186/s13020-022-00682-9

**Published:** 2022-11-08

**Authors:** Yanyan Shi, Jing Ning, Kelsang Norbu, Xingzi Hou, Huiling Zheng, Hejun Zhang, Wei Yu, Feng Zhou, Yuan Li, Shigang Ding, Qingying Zhang

**Affiliations:** 1 Research Center of Clinical Epidemiology, Peking University Third Hospital, Beijing, 100191 People’s Republic of China; 2 Department of Gastroenterology, Peking University Third Hospital, No. 49, Garden North Road, Haidian District, Beijing, 100191 People’s Republic of China; 3 Department of Research and Development, Tibet Ganlu Tibetan Medicine Co., Ltd, Lhasa, 850000 People’s Republic of China; 4 Department of Natural Medicines, School of Pharmaceutical Sciences, Peking University Health Science Center, No. 38, Xueyuan Road, Haidian District, Beijing, 100191 People’s Republic of China; 5 Department of Gastroenterology, Affiliated Hospital of Jining Medical University, Jining, 272000 People’s Republic of China

**Keywords:** Tibetan medicine, Zuozhu-Daxi, *Helicobacter pylori*, Gastric mucosal injury, Gastric mucosal inflammation

## Abstract

**Background:**

Tibetan medicine has been used in clinical practice for more than 3800 years. Zuozhu-Daxi (ZZDX), a classic traditional Tibetan medicine, has been proved to be effective in the treatment of digestive diseases, such as chronic gastritis, gastric ulcer, etc. *Helicobacter pylori* (*H. pylori*), one of the most common pathogenic microbes, is regarded as the most common cause of gastritis. Researching on the effects of ZZDX on *H. pylori*-induced gastric mucosa inflammation could provide more evidences on *H. pylori* treatment and promote the development of Tibetan medicine. This study aimed to explore whether ZZDX could rescue *H. pylori*-induced gastric mucosa inflammation and its mechanism.

**Methods:**

Male C57BL/6 mice were infected with *H. pylori*, and orally treated with ZZDX to rescue gastric mucosa inflammation induced by *H. pylori* infection. Pathology of gastric mucosa inflammation was evaluated under microscopy by hematoxylin–eosin (HE) staining. The infection status of *H. pylori* was evaluated by immunohistochemical (IHC) staining. The reactive oxygen species (ROS) level in serum was evaluated using a detection kit. IL-1α, IL-6, and PGE2 expression levels in serum were measured using ELISA. IL-1α, IL-8, TNF-α, and NOD1 expression levels in gastric tissues were measured using real-time PCR. RNA sequencing and gene certification of interest were performed to explore the mechanisms in vivo and in vitro.

**Results:**

The results showed that ZZDX could significantly inhibit *H. pylori*-induced gastric mucosa inflammation using HE staining. IL-1α, IL-6, and PGE2 expression levels in serum were significantly decreased after treatment with ZZDX. ZZDX treatment significantly decreased the mRNA expression of IL-8 induced by *H. pylori* infection in gastric tissues. *Elovl4, Acot1* and *Scd1* might be involved in the mechanisms of ZZDX treatment. However, the *H. pylori* infection status in the gastric mucosa was not reduced after ZZDX treatment.

**Conclusions:**

ZZDX reversed gastric mucosal injury and alleviated gastric mucosa inflammation induced by *H. pylori* infection.

## Background

*Helicobacter pylori* (*H. pylori*), a gram-negative bacterium which colonizes approximately 50% of the population worldwide [1], is one of the most common pathogenic microbes and is regarded as the major cause of gastritis. It is well known that chronic infection of *H. pylori* can even lead to gastric precancerous lesions including mucosal atrophy and intestinal metaplasia, and thus long-term infection ultimately can result in gastric cancer [2]. In 1994, *H. pylori* was defined as a class I carcinogen by the world health organization (WHO) [3]. Patients with gastric precancerous lesions that do not to be reversed are considered to be at high risk for gastric cancer development. Therefore, effective remedies for *H. pylori*-induced gastric mucosa inflammation should be improved to prevent gastric cancer development [4], and updated therapies are urgently needed to effectively suppress *H. pylori-* or *H. pylori*-induced gastric mucosa inflammation [5]. At present, there have been many studies on the effects of *H. pylori* infection treated by traditional Chinese medicine (TCM) [6]. The fifth Chinese national consensus report on *H. pylori* infection management has proposed that TCM and proprietary Chinese medicines were worthy to be validated for *H. pylori* treatment [7, 8]. TCM, for example Banxia Xiexin decoction, has been proved to be effective in reducing drug resistance and increasing *H. pylori* eradication rate [9].

Tibetan medicine has a long history of 3800 years spanning from the sixth century Anno Domini (A. D.) [10]. Zuozhu-Daxi (ZZDX) is a classic traditional Tibetan medicine, which composing of Calcite Lactis Praeparata, Calciosinti, Bambusae Concretio Silicea, Herba Aconiti Tangutici, Croci Stigma, Myristicae Semen, Tsaoko Fructus, Carthami Flos, Pulvis Fellis Ursi, Artificial Bovis Calculus, Artificial Moschus, etc., as shown in Table 1. ZZDX, possessing the efficacy of calming the liver, invigorating the stomach, clearing heat, curing anabrosis and relieving swellness [11], has a more than 600-year history of practical application. In Tibetan hospitals, ZZDX has been used for the treatment of liver pain, indigestion, “Huangshui” disease, visceral tumors, and food poisoning. Particularly, it has been widely used for digestive diseases, such as gastrohelcosis, duodenal ulcer, chronic gastritis, and gastric cancer [12]. In the current study, we elucidated the effects and potential mechanisms of ZZDX on gastric mucosa inflammation in vivo and in vitro using *H. pylori* infected mice and gastric epithelial cell lines.

**Table 1:** Detailed information of Tibetan medicine Zuozhu-Daxi

Medicinal material name (*In Chinese*)	Origin	Medicinal part, processing	Major constituent	Analysing method	Analysed result	Medicinal material No
Calcite Lactis Praeparata (Bei *Hanshuishi(Naizhi)*)	/	Sulfate minerals, remove impurities and mix with milk to make a paste	CaSO_4_	/	/	Y-21041201
Calciosinti (*Shihuihua*)	*/*	Carbonate minerals, remove impurities	CaCO_3_	/	/	Y-11070701
Bambusae Concretio Silicea (*Tianzhuhuang*)	*Bambusae textilis* MuClure	Secretion in culm, dried	Leucine, Alanine	TLC (365 nm)	Exhibits the bands corresponding in color and R_f_ similar to those in the chromatogram of standard medicinal materials	Y-19050501
Aconiti naviculare Herba (*Chuanxingwutou*)	*Aconitum naviculare* (Bruhl.) Stapf	Whole herb, dried	/	/	/	Y-21112302
Croci Stigma (*Xihonghua*)	*Crocus sativus* L	Flower, dried	Crocin-I, Crocin-II, Crocin	HPLC–UV (440 nm, 254 nm)	Sum content of Crocin-I and Crocin-II and content of crocin is 11.8% and 6.7%, respectively	Y-22042703
Myristicae Semen (*Roudoukou*)	*Myristicae fragrans* Houtt	Fruit, dried	Volatile oil, Dehydrodiisoeugenol	Steam distillation, HPLC–UV (274 nm)	Content of volatile oil and dehydrodiisoeugenol is 6.8% and 0.28%, respectively	Y-22051604
Tsaoko Fructus (*Caoguo*)	*Amomum tsaoko* Crevost et Lemaire	Fruit, dried	Volatile oil	Steam distillation	Content of volatile oil is 1.53%	Y-13101504
Carthami Flos (*Honghua*)	*Carthamus tinctorius* L	Flower, dried	Hydroxysafflor yellow A, Kaempferol	HPLC–UV (403 nm, 367 nm)	Content of hydroxysafflor yellow A and kaempferol is 1.48% and 0.099%, respectively	Y-13040804
Pulvis Fellis Ursi (*Xiongdan Fen*)	*Selenartos thibetanus* G. Cuvier	Bile, dried	/	/	/	Y-20011402
Artificial Bovis Calculus (*Rengong Niuhuang*)	Synthetic	/	Cholic acid, Bilirubin	TLC (λ_S_ = 380 nm, λ_R_ = 650 nm), HPLC–UV (450 nm)	Content of cholic acid and bilirubin is 13.5% and 0.7%, respectively	Y-21120101
Artificial Moschus (*Rengong Shexiang*)	Synthetic	/	Muscone	HPLC-GC	Content of muscone is 2.7%	P-20122801
Others	Except for the 11 prescription drugs mentioned above, the rest others are not public					

## Materials and methods

### Zuozhu-Daxi (ZZDX)

ZZDX was provided by Tibet Ganlu Tibetan Medicine Co., Ltd., and its detailed information is shown in Table 1. After grinding, ZZDX was suspended in phosphate-buffered saline (PBS) to appropriate concentration for in vivo and in vitro experiments.

### *H. pylori* culture

*H. pylori* strains ATCC 26,695 and SS1 were obtained from the Key Laboratory for *Helicobacter pylori* Infection and Upper Gastrointestinal Diseases in Peking University Third Hospital, and the strains ATCC 26,695 and SS1 were cultured on blood agar plates containing 39 g/L Columbia solid culture medium (Oxoid), 5% (v/v) sheep’s blood (Curtin Matheson, Jessup, MD, USA) supplemented with antibiotics amphotericin B (4 μg/mL) (Life Tech), trimethoprim (4 μg/mL) and vancomycin (4 μg/mL). The plates were incubated in a microaerobic environment [5% (v/v) O_2_, 10% (v/v) CO_2_ and 85% (v/v) N_2_] at 37 °C. *H. pylori* were harvested directly from 24- to 48-h plate cultures. *H. pylori* strains were examined before harvesting to be confirmed through Gram staining, urease tests, oxidase tests and catalase tests.

### *H. pylori*-infected animal models and ZZDX treatment

A total of twenty-four six-week-old male specific pathogen free (SPF) level C57BL/6 mice were purchased from the China National Institute for Food and Drug Control (Daxing) Animal Resource Center and kept in an air-conditioned and barrier environment. These twenty-four mice were divided into four groups. Group 1 was the negative control (NC) group, which was intubated with *Brucella* broth alone. Group 2 was the *H. pylori*-infected (HP) group, and every mouse was intubated five times with 0.5 ml *Brucella* broth of *H. pylori* SS1 containing 3 × 10^8^ CFU/mL. Group 3 was the low-dose ZZDX-treated (HP + ZZDX low-dose) group, with 0.083 g/kg ZZDX treatment for seven days after *H. pylori* infection. Group 4 was the high-dose ZZDX-treated group (HP + ZZDX high-dose), with 0.166 g/kg ZZDX treatment for seven days after *H. pylori* infection. Subsequently, the mice were killed by cervical dislocation. Blood and gastric tissues were processed and collected for further analyses.

The reasoning for our choice of ZZDX dose was based on the clinical dose of ZZDX. In clinical practice, the recommended daily dose of ZZDX was 1000 mg per person (60 kg weight), i.e., 8.3 mg/kg every day. According to the body surface area method in pharmacology, the dose used in mice should be approximately ten times the dose used in humans. Therefore, the daily dose of ZZDX in mouse models should be 83 mg/kg. In this study, two doses, 0.083 g/kg/day and 0.166 g/kg/day, were investigated.

### Histopathological analysis

The dissected gastric tissues along the greater curvature were washed with PBS, fixed in paraformaldehyde, embedded in paraffin, and sliced into 3 μm sections. Each specimen was stained by hematoxylin–eosin (HE) to evaluate the pathology of gastric mucosa inflammation under microscopy. Moreover, immunohistochemical (IHC) testing was used to evaluate the infection status of *H. pylori* (*H. pylori* Antibody Reagent for Immunohistochemistry, ZSGB-BIO, Beijing, China) and the expression levels of proteins. The expression levels of ACOT1 and ELOVL4 (polyclonal rabbit anti-human antibodies at a concentration of 1:1000, ImmunoWay Biotechnology Company, Texas, USA) were detected by IHC testing following the manufacturer’s instructions. Histopathological analysis was performed independently by two experienced pathologists.

### Reactive oxygen species (ROS) measurement

ROS levels in serum were evaluated using a detection kit (BBoxiProbe O12 ROS, BestBio, Shanghai, China). Briefly, 10 μL of O12 probe diluted tenfold in ddH_2_O was added to 100 μL fresh serum and incubated at 37 ℃ for 30 min in the dark. The fluorescence intensity was detected at an excitation wavelength of 488 nm and an emission wavelength of 530 nm.

### ELISA analysis

IL-1α, IL-6, and PGE2 expression levels in animal serum and cell culture supernatant were measured using an ELISA kit (MLBio, Shanghai, China) following the manufacturer’s instructions.

### RNA extraction and real-time PCR analysis

Total RNA from tissue and cells was extracted using TRIzol (Invitrogen, Shanghai, China). RNA was reverse-transcribed into cDNA using the Super-Script First-Strand cDNA System (Invitrogen, Carlsbad, CA, USA), and real-time qPCR monitoring of cDNA was performed using the Roche LightCycler 480 sequence detection system (Roche, Mannheim, USA). Beta-actin (Actb) was used as an internal reference gene, and the primers used for RT-qPCR are shown in Table 2 below.

**Table 2:** Primer sequences used for RT-qPCR

Primer	Sequence(5'-3')
*Il1a* qF (Mus)	CGAAGACTACAGTTCTGCCATT
*Il1a* qR (Mus)	GACGTTTCAGAGGTTCTCAGAG
*Cxcl15* qF (Mus)	CAAGGCTGGTCCATGCTCC
*Cxcl15* qR (Mus)	TGCTATCACTTCCTTTCTGTTGC
*Tnf* qF (Mus)	CCCTCACACTCAGATCATCTTCT
*Tnf* qR (Mus)	GCTACGACGTGGGCTACAG
*Scd1* qF (Mus)	TTCTTGCGATACACTCTGGTGC
*Scd1* qR (Mus)	TTGAGCCTTTGTAAATGGGCA
*Acot1* qF (Mus)	ATACCCCCTGTGACTATCCTGA
*Acot1* qR (Mus)	CAAACACTCACTACCCAACTGT
*Acot2* qF (Mus)	GTTGTGCCAACAGGATTGGAA
*Acot2* qR (Mus)	GCTCAGCGTCGCATTTGTC
*Elovl4* qF (Mus)	GTCCTGAACGCGATGTCCA
*Elovl4* qR (Mus)	GCGTGCTTATGCTTATCGTTG
*Nod* qF (Mus)	TGTCAGGATCTCGCATTGGT
*Nod* qR (Mus)	ATTGCTTCGTAGATAGAGGTGTGTG
*IL1A* qF (Homo)	AGATGCCTGAGATACCCAAAACC
*IL1A* qR (Homo)	CCAAGCACACCCAGTAGTCT
*CXCL8* qF (Homo)	ACTGAGAGTGATTGAGAGTGGAC
*CXCL8* qR (Homo)	AACCCTCTGCACCCAGTTTTC
*TNF* qF (Homo)	GAGGCCAAGCCCTGGTATG
*TNF* qR (Homo)	CGGGCCGATTGATCTCAGC
*SCD* qF (Homo)	TTCCTACCTGCAAGTTCTACACC
*SCD* qR (Homo)	CCGAGCTTTGTAAGAGCGGT
*ACOT2* (Homo) qF	CGTCCCGGCTGTACCAATG
*ACOT2* (Homo) qR	GGAACCCTAATGATCTGACCAAC
*ELOVL4* qF (Homo)	AAGGACCGAGAACCTTTTCAGA
*ELOVL4* qR (Homo)	TCCCGCATTATATGATCCCATGA
*ACOT1* qF (Homo)	TGCTGGAGTATCGGGCTAGT
*ACOT1* qR (Homo)	ACCTCAGGATGACTGAGCAAG
*ACTB* qF (Homo)	TTGTTACAGGAAGTCCCTTGCC
*ACTB* qR (Homo)	ATGCTATCACCTCCCCTGTGTG
*Actb* qF (Mus)	GGCTGTATTCCCCTCCATCG
*Actb* qR (mus)	CCAGTTGGTAACAATGCCATGT

### RNA sequencing for mouse gastric tissue

Total RNA was extracted from mouse gastric tissue using TRIzol according to the manufacturer's protocol. The RNA quality was checked by a Bioanalyzer 2100 (Agilent, USA), and the integrity number (RIN) of all the RNA samples was > 9.0.

The sequencing libraries were prepared using the Illumina TruSeqTM RNA Sample Prep Kit. Briefly, poly-A-containing mRNA was isolated from the total RNA by poly-T oligo-attached magnetic beads. cDNA was synthesized using random primers through reverse transcription. After ligation with the adaptor, the cDNA was amplified by 15 cycles of PCR, and then 200-bp fragments were isolated using gel electrophoresis. Finally, the products were sequenced by an Illumina NovaSeq 6000 instrument at Majorbio Co., Ltd. (China). The raw data have been submitted to the NCBI Gene Expression Omnibus (GEO) database under accession number GSE.

After sequencing, the screening of DEGs was based on their TPM (transcripts per kilobase million) values. A false discovery rate (FDR) of 0.05 and an absolute value of log2FC > 1 were used to identify significant DEGs. To inspect the functions of DEGs, GO enrichment analysis and KEGG pathway enrichment analysis of the DEGs were performed.

### Cell culture, co-culture assays and ZZDX treatment

Human gastric epithelial GES-1 cells were cultured in Roswell Park Memorial Institute (RPMI) 1640 medium supplemented with 10% (v/v) fetal bovine serum (FBS) (PAN-Biotech, Adenbach, Germany) at 37 °C in a humidified incubator at 5% (v/v) CO_2_. For co-culturing of cells and strains, first, *H. pylori* 26,695 were harvested from 24- to 48-h plate cultures, washed with PBS three times, and resuspended in cell growth medium and diluted to a final concentration of 1 × 10^8^ CFU/mL. Then, GES-1 cells were plated one day before *H. pylori* treatment and rinsed once with PBS before fresh growth medium was added. Finally, the diluted bacterial strains were added to the cell medium at multiplicities of infection (MOIs) of 100:1. Zuozhu-Daxi was added to the co-cultured cells at concentrations of 20 μg/mL, 50 μg/mL, 100 μg/mL and 200 μg/mL. Uninfected GES-1 cells were negative controls. Cells co-cultured only with *H. pylori* were positive controls.

### Western blot analysis

Proteins related to the PPAR signalling pathway were detected by Western blot analysis. Harvested cells were lysed in cell lysis buffer containing protease inhibitors for 30 min on ice. Then, the cell lysate was centrifuged at 15 000 × g at 4 °C for 10 min, and the supernatant was collected. The total protein concentration was measured by a bicinchoninic acid (BCA) protein assay kit (Thermo Scientific, Shanghai, China). 10% (w/v) SDS-PAGE was used to separate proteins, and then electrophoretically transferred proteins onto PVDF membranes. The membranes were blocked in 5% (w/v) fat-free milk in PBS supplemented with 0.1% (v/v) Tween-20 at room temperature for 1 h. After blocking, the membranes with proteins were incubated overnight at 4 °C with antibodies against ELOVL4 (polyclonal rabbit anti-human antibody, Proteintech, Rosemont, USA), ACOT1 (polyclonal rabbit anti-human antibody, Abcam, Shanghai, China), SCD1 (monoclonal rabbit anti-human antibody, Abcam, Shanghai, China), PPAR (polyclonal mouse anti-human antibody, ImmunoWay Biotechnology Company, Texas, USA) and β-actin (polyclonal rabbit anti-human antibody, CST, Shanghai, China). After being washed three times for 10 min each in PBS supplemented with 0.1% (v/v) Tween-20, the membranes were incubated with a secondary antibody for 1 h at room temperature. Then, the membranes were washed as in the previous step, and protein bands were scanned by an Odyssey Imager (LI-COR Biosciences).

### Statistical analysis

Data were presented as the mean ± s.d. of three independent experiments. The differences among more than two groups were analysed using one-way ANOVA. The differences between two groups were analysed using Student’s t test. All statistical analyses were performed using SPSS 23.0 software. *P* values < 0.05 were considered statistically significant.

## Results

### ZZDX treatment reversed the gastric mucosa injury induced by H. pylori but did not decrease *H. pylori* colonization in mouse gastric mucosa

**Fig. 1:**
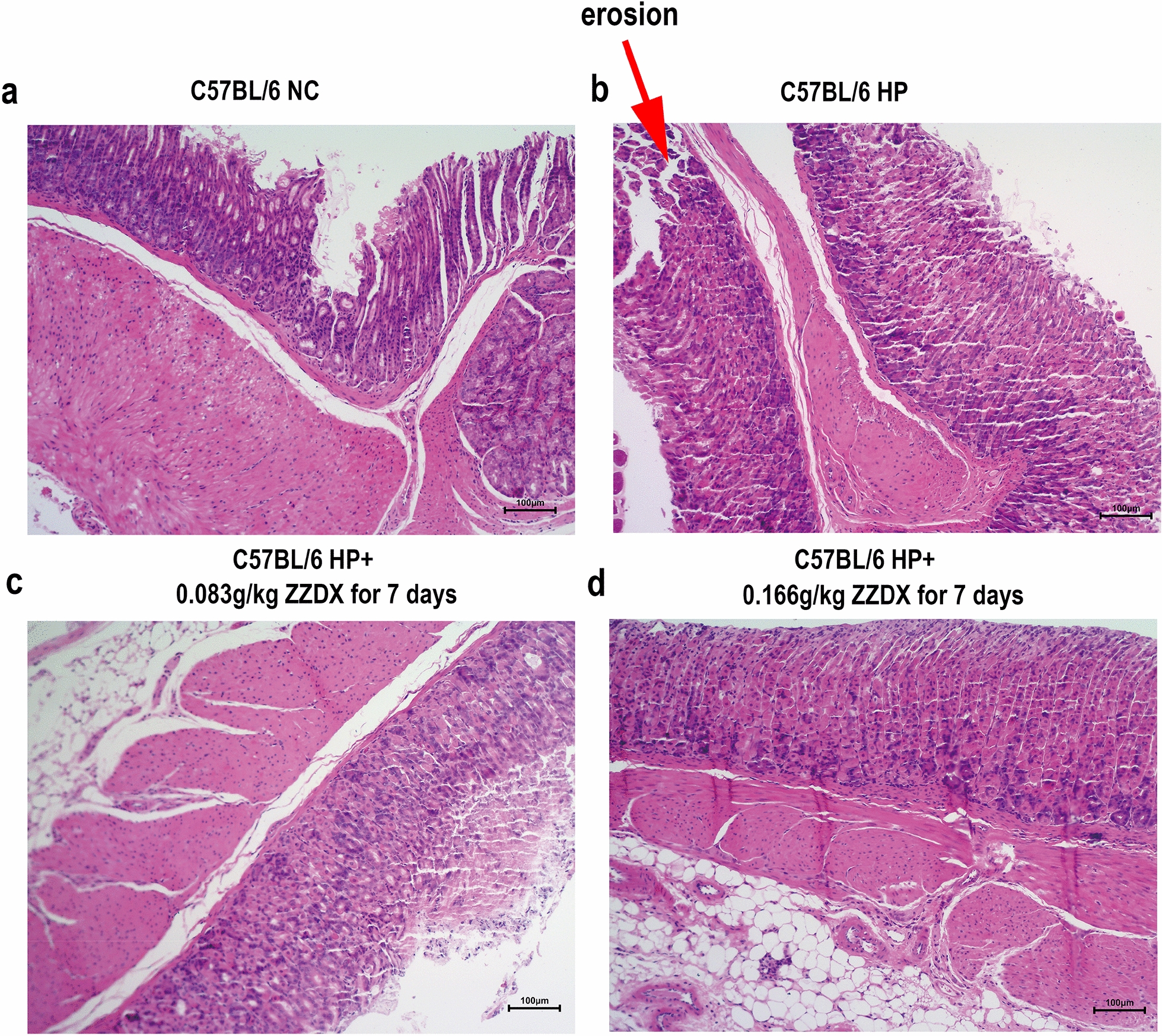
ZZDX treatment reversed the gastric mucosa injury induced by *H. pylori*. HE staining showed that the gastric mucosa of the NC group was normal (**a**), and it was injured and showed erosion in the HP group (**b**). After ZZDX treatment at either the low dose (**c**) or the high dose (**d**), the gastric mucosa injury caused by *H. pylori* infection was reversed to a certain extent

**Fig. 2:**
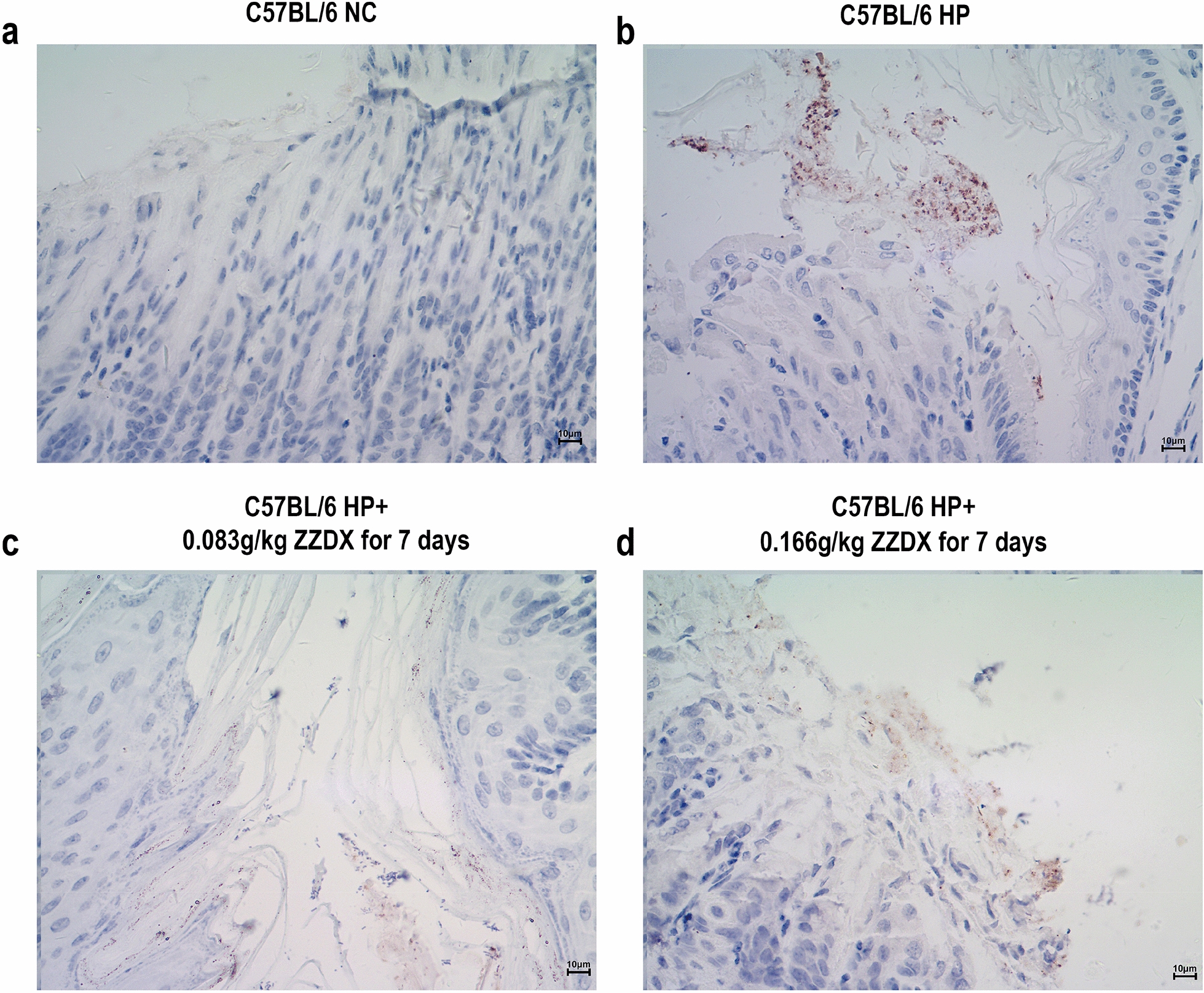
ZZDX treatment did not decrease *H. pylori* colonization in mouse gastric mucosa. *H. pylori* immunohistochemical staining showed that compared with the NC group (**a**), *H. pylori* was successfully colonized in the HP group (**b**). After ZZDX treatment at either the low dose (**c**) or the high dose (**d**), the colonization of *H. pylori* in mouse gastric mucosa was not decreased

HE staining was used to evaluate the pathology of gastric mucosa inflammation under microscopy. Our results showed that the *H. pylori*-infected gastric mucosa inflammation mouse model was successfully established. In Fig. 1, the gastric mucosa of the NC group was normal (Fig. 1a). In the HP group, the gastric mucosa was injured and showed erosion (Fig. 1b). After ZZDX treatment, the gastric mucosa injury caused by *H. pylori* infection could be reversed to a certain extent (Fig. 1c, d). To gain further insight into the status of *H. pylori* in gastric mucosa, we assayed *H. pylori* colonization in mouse gastric mucosa using *H. pylori* immunohistochemical staining, and the results showed that *H. pylori* was successfully colonized in the HP group (Fig. 2a, b). However, the colonization of *H. pylori* in mouse gastric mucosa was not decreased after ZZDX treatment (Fig. 2c, d).

### ZZDX treatment decreased the expression levels of inflammatory factors induced by *H. pylori* infection

**Fig. 3:**
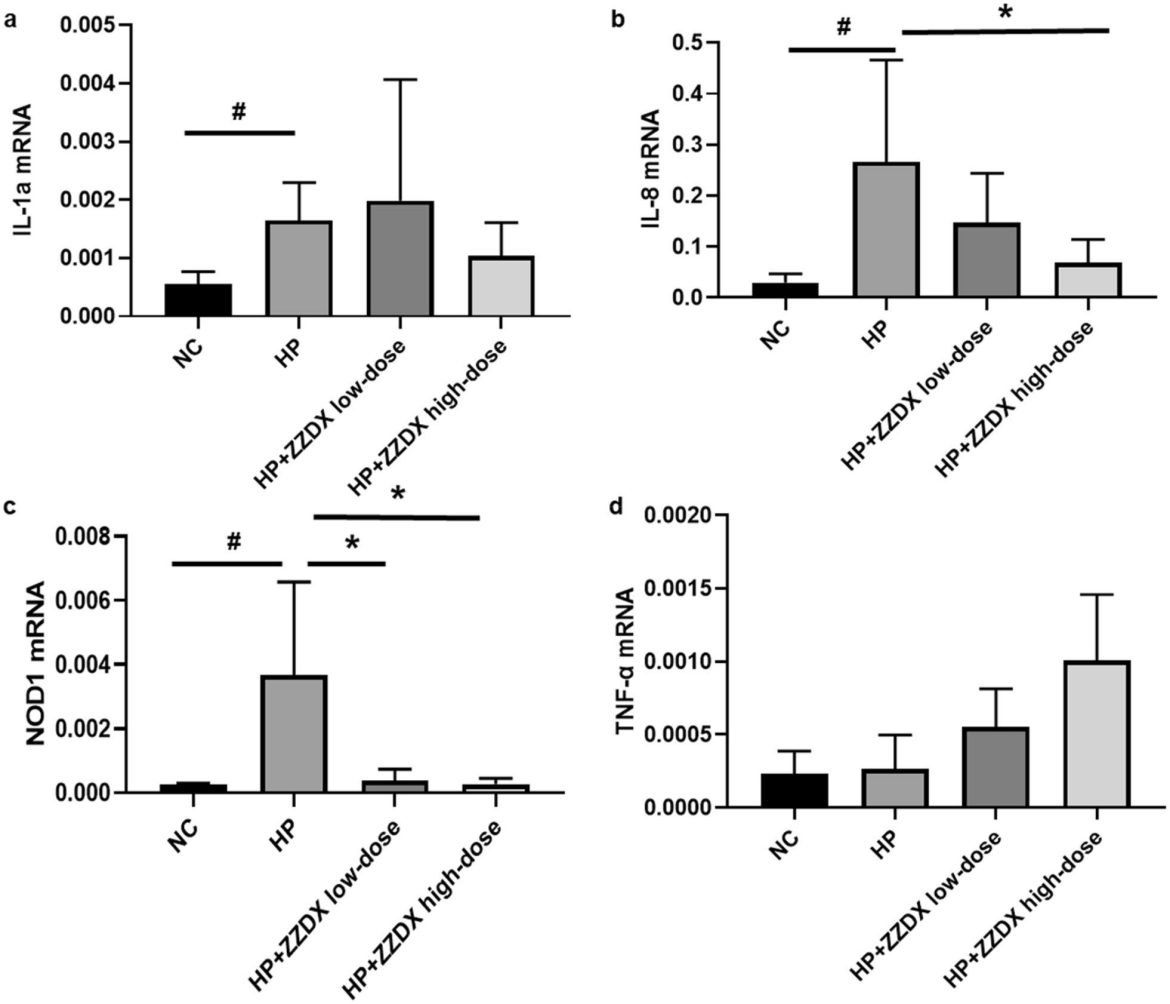
ZZDX treatment decreased the mRNA levels of inflammatory factors induced by *H. pylori* infection. The mRNA levels of IL-8 (**b**) and NOD1 (**c**) were significantly downregulated after ZZDX treatment at the high dose, while the mRNA levels of IL-1α (**a**) and TNF-α (**d**) were found not to be significantly decreased. ^**#**^*P* < 0.05 vs. NC group; *P < 0.05 vs. HP group

**Fig. 4:**
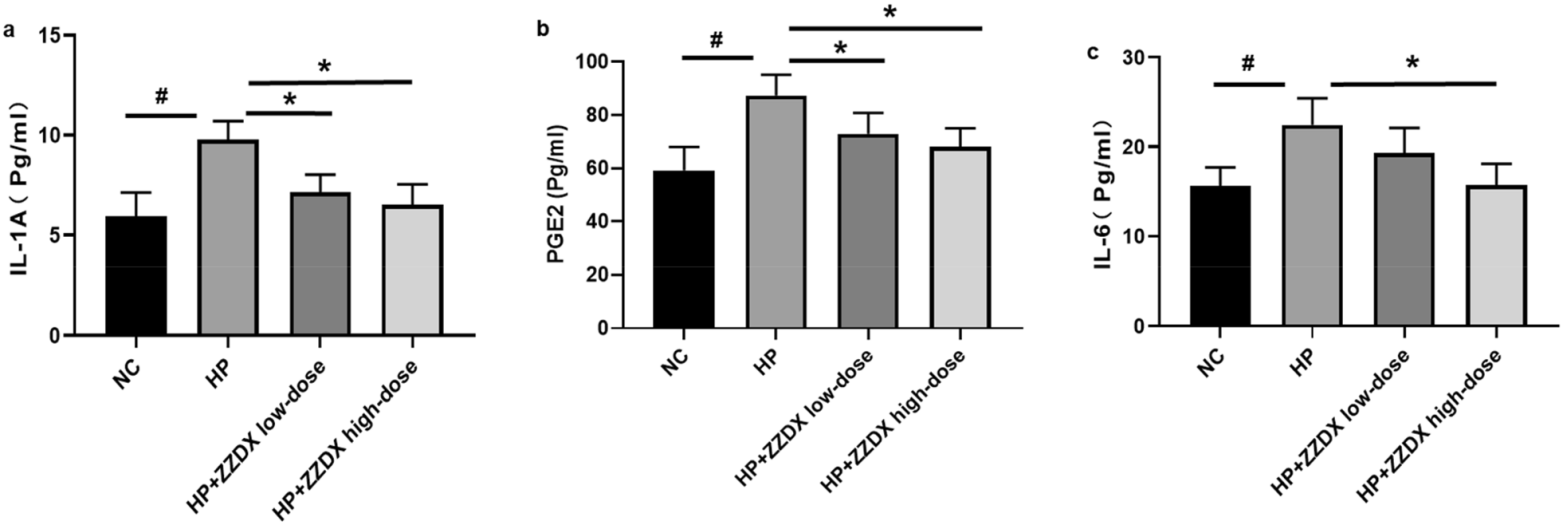
ZZDX treatment decreased the serum expression levels of inflammatory factors induced by *H. pylori* infection. The expression levels of IL-1α (**a**) and PGE2 (**b**) were significantly downregulated after ZZDX treatment at either the low dose or the high dose in a dose-dependent manner. The expression levels of IL-6 (**c**) was significantly downregulated after ZZDX treatment at the high dose. ^**#**^*P* < 0.05 vs. NC group; *P < 0.05 vs. HP group

To examine whether ZZDX could decrease the expression levels of inflammatory factors induced by *H. pylori* infection, real-time PCR for gastric mucosa tissues and ELISA for serum were used to measure the levels of inflammatory factors. The real-time PCR results for gastric mucosa tissues showed that *H. pylori* infection could significantly increase the mRNA levels of IL-1α, IL-8 and NOD1 (*P* < 0.05), but no change was found for TNF-α. After ZZDX treatment at the high dose of 0.166 g/kg, the mRNA level of IL-1α in gastric mucosa was downregulated, with no significant difference (Fig. 3a), and the mRNA levels of IL-8 and NOD1 in gastric mucosa were downregulated significantly (Fig. 3b, c). The ELISA results showed that *H. pylori* infection significantly upregulated the levels of IL-1A, IL-6 and PGE2 in the serum of the mouse model (Fig. 4). After ZZDX treatment at either the low dose of 0.088 g/kg or the high dose of 0.166 g/kg, IL-1A and PGE2 were decreased in a dose-dependent manner (Fig. 4a, b), while IL-6 was reversed significantly at the high dose (Fig. 4c).

### Exploring the anti-inflammatory mechanisms of ZZDX on *H. pylori*-infected gastric mucosa using RNA sequencing

**Fig. 5:**
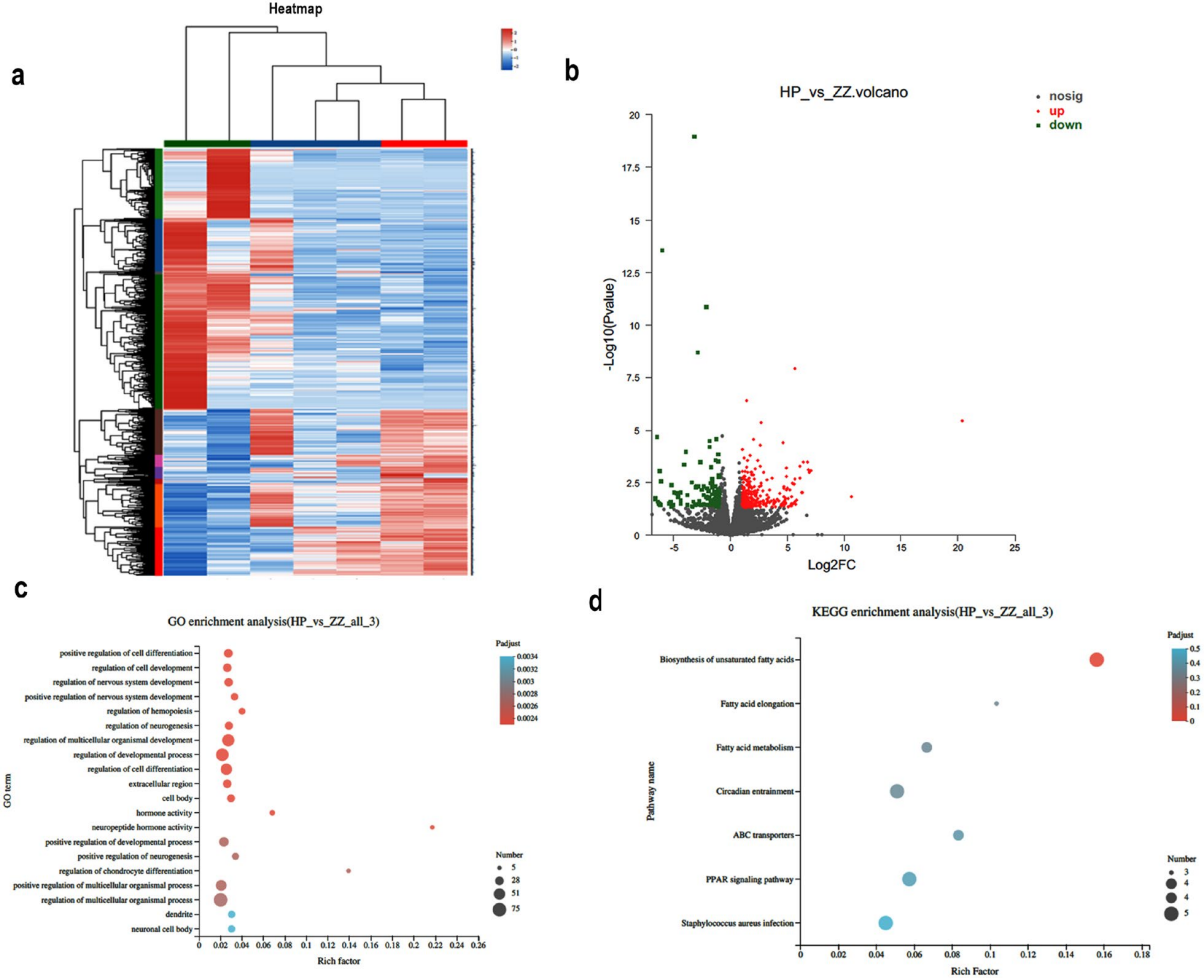
RNA sequencing of mouse gastric mucosa from the three groups showed that 401 genes were regulated by ZZDX treatment. **a** Heatmap of differentially expressed genes in the NC group, HP group and ZZDX group. **b** A volcano plot was constructed from the differentially expressed genes between the HP group and ZZDX group, including 119 downregulated genes and 282 upregulated genes. **c** Gene Ontology (GO) enrichment of differentially expressed genes was performed, and the top 20 enriched GO terms were shown. **d** KEGG pathway analysis of genes regulated by ZZDX treatment was performed

**Fig. 6:**
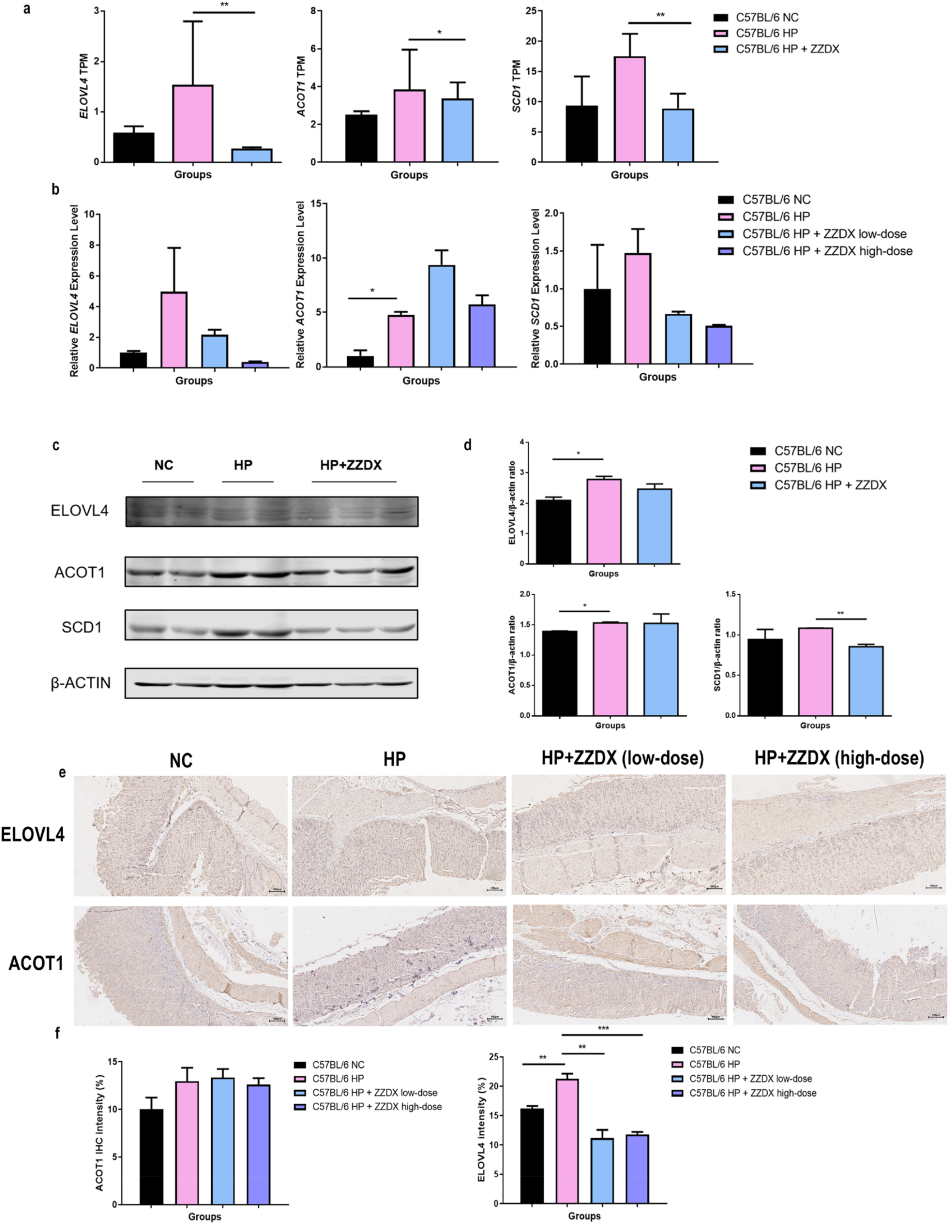
ZZDX affected the genes expression that related to the unsaturated fatty acid biosynthesis pathway. **a** The mRNA expression levels of Elovl4, Acot1 and Scd1 were significantly decreased after ZZDX treatment. **b** RT-qPCR validaton results of differentially expressed genes. **c** and (**d**) The western blot results of mouse gastric mucosa and its grayscale analysis results showed the same result with RT-qPCR. **e** and (**f**) The IHC results of mouse gastric mucosa and its staining intensity analysis also showed that after *H. pylori* infection, the expression levels of ELOVL4 and ACOT1 were upregulated in situ and significantly decreased after ZZDX treatment. ^**#**^*P* < 0.05; ^**##**^*P* < 0.01 vs. NC group; *P < 0.05; **P < 0.01; ***P < 0.001 vs. HP group

To explore the potential anti-inflammatory molecular mechanisms of ZZDX on *H. pylori*-infected gastric mucosa, RNA sequencing was used to analyse the differentially expressed genes among groups, including 2 mice from the NC group, 2 mice from the HP group, and 3 mice from the ZZDX-treated groups. The expression levels of IL-8 in the NC group were 0.020 and 0.051, 0.391 and 0.534 in the HP group and 0.037, 0.136, and 0.024 in the ZZDX-treated group at the high dose of 0.166 g/kg. The histopathology of the mucosa in the above three groups was normal, chronic gastritis and normal, respectively. A heatmap was constructed from the data obtained for the differentially expressed genes (Fig. 5a). Between the NC group and the HP group, 2596 genes were identified to be differentially expressed significantly. Between HP groups with or without ZZDX treatment, 401 genes were identified to be differentially expressed significantly, including 119 downregulated genes and 282 upregulated genes. A volcano map was constructed from the differentially expressed genes between the HP groups with or without ZZDX treatment (Fig. 5b). Gene Ontology (GO) enrichment of differentially expressed genes was performed, and the top 20 enriched GO terms were shown in Fig. 5c according to the *P* values of the enriched GO terms. The top four most enriched GO terms were “positive regulation of cell differentiation”, “regulation of cell development”, “regulation of nervous system development”, and “positive regulation of nervous system development”. KEGG pathway analysis of genes regulated by ZZDX treatment was shown in Fig. 5d, indicating that the differentially expressed genes regulated by ZZDX were most enriched in “Biosynthesis of unsaturated fatty acids”, “Fatty acid elongation”, “Fatty acid metabolism”, and “Circadian entrainment”. Genes such as acyl-CoA thioesterase 1 (ACOT1), ELOngation of Very Long-chain fatty acid-4 (ELOVL4), stearoyl-CoA desaturase 1 (SCD1) and peroxisome proliferator activated receptor gamma (PPARG) were included in the prominent pathway “biosynthesis of unsaturated fatty acids”, and their expression levels were significantly affected by *H. pylori* infection and drug administration (Fig. 6a).

### Verification of four identified proteins

In support of the above results, RT-qPCR and western blot analysis were conducted to monitor changes in the levels of four identified genes implicated in “biosynthesis of unsaturated fatty acids” (Fig. 6b–d). As shown in the results, the expression levels of ELOVL4, ACOT1 and SCD1 increased when *H. pylori* was infected and decreased after ZZDX treatment. The IHC staining results of mouse gastric mucosa also showed that the expression levels of ELOVL4 and ACTO1 were upregulated in situ after *H. pylori* infection and significantly decreased after ZZDX treatment (Fig. 6e, f).

**Fig. 7:**
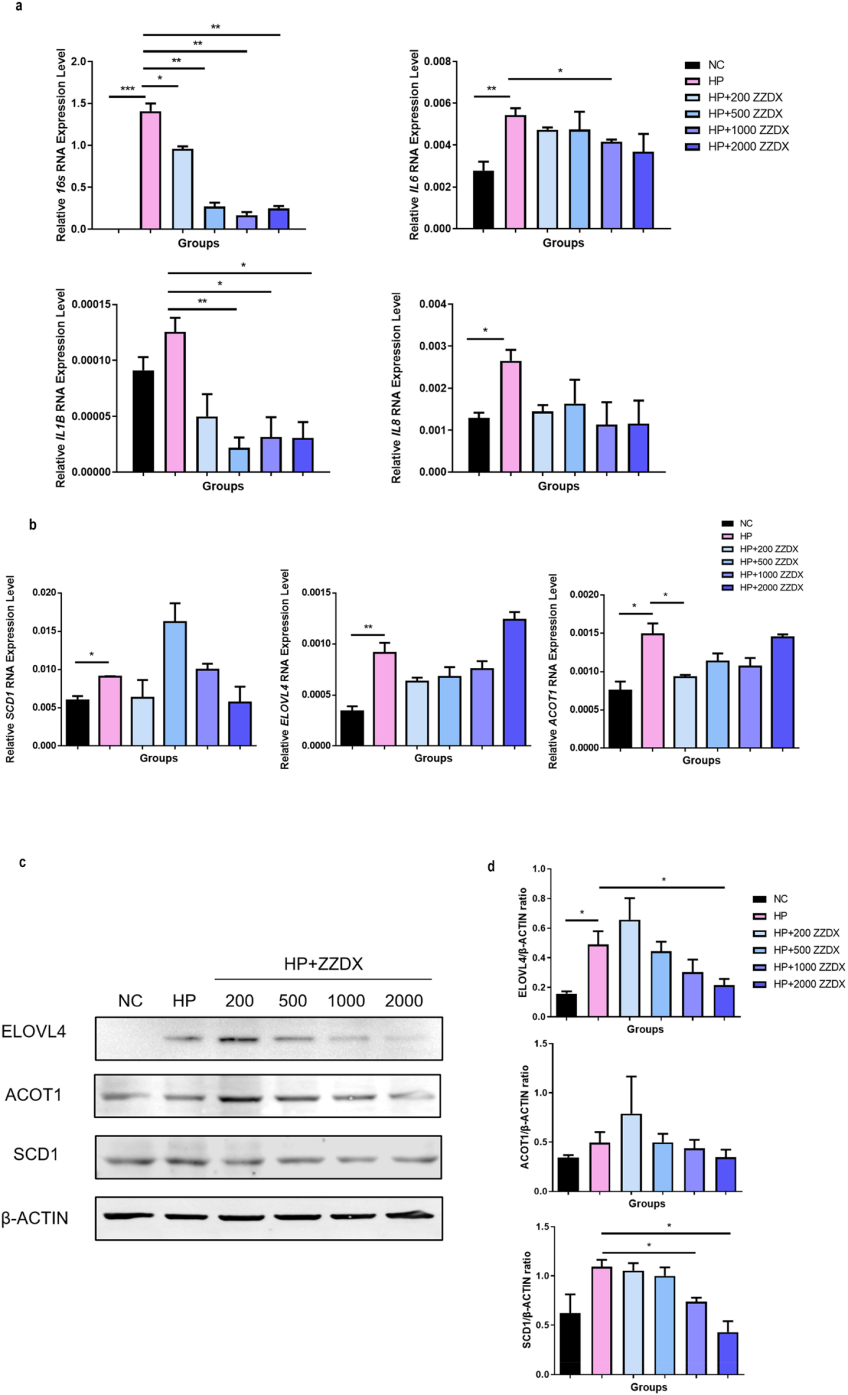
ZZDX showed the same effect in cell line experiments. **a** The RT-qPCR results showed that ZZDX decreased the expression levels of IL-1, IL-6 and IL-8 and decreased the number of *H. pylori* attached to cells. **b** The mRNA expression levels of Elovl4, Acot1 and Scd1 were significantly decreased after ZZDX treatment in vitro. **c** and (**d**) The western blot results of cells and their grayscale analysis results showed the same result with RT-qPCR. N = 3. ^**#**^*P* < 0.05; ^**##**^*P* < 0.01; ^**###**^*P* < 0.001 vs. NC group; *P < 0.05; **P < 0.01 vs. HP group

Furthermore, the above results were verified in vitro. *H. pylori* infection significantly increased the mRNA levels of IL-6 and IL-8, and the mRNA level of IL-1 was also increased. After ZZDX treatment, the mRNA levels of IL-1 and IL-6 were downregulated significantly (Fig. 7a). The expression levels of ELOVL4, SCD1 and ACOT1 before and after ZZDX treatment were further verified by RT-qPCR and western blot, which were consistent with the results above (Fig. 7b, c). These results suggested that ZZDX can effectively inhibit the increase in lipid metabolism and inflammation caused by *H. pylori* infection, thereby effectively alleviating the occurrence and development of gastritis and gastric mucosal diseases caused by *H. pylori* infection.

## Discussion

It is estimated that more than half of the world’s population are infected with *H. pylori* [1]. As a class I carcinogen by the WHO, the carcinogenesis induced by *H. pylori* from chronic gastritis to ultimately gastric cancer is a multi-step and multi-level process [13, 14]. To reduce the incidence of gastric cancer, eradication of *H. pylori* is the main therapeutic strategy by the combination of antibiotics and proton pump inhibitors. However, it poses a huge challenge to the eradication therapy of *H. pylori* owing to undesired side effects as well as the emergence of steadily increasing antibiotic-resistant strains [15].

In recent years, traditional medicine has become a source of new pharmaceuticals due to their strong efficacy with fewer side effects and lower toxicity, and have made surprising progress in the treatment of various diseases, such as tumors, inflammation, gout, atherosclerosis, virus infection, bacterial infection and fungal infection [16–22]. Tibetan medicine, as an important traditional medicine, has unique advantages in the treatment of peptic ulcers. Of which, ZZDX is mainly applied for the treatment of chronic gastritis, peptic ulcer and gastric cancer, and shows a potential reversal effect on gastric mucosal damage [23, 24].

In this study, mice were orally treated with ZZDX to rescue gastric mucosa inflammation induced by *H. pylori* infection, and the infection status of *H. pylori* was also measured in the mouse gastric mucosa. The results showed that ZZDX might have a reversal effect on the inflammation of gastric mucosa induced by *H. pylori* infection. According to the literature reports, IL-1α [25], IL-8 [26], TNF-α [27], and NOD1 [28] play an important role in the *H. pylori* bacterial infection process and gastric mucosal inflammation and promote the synthesis and release of other cytokines. After *H. pylori* infection, the immune system can be activated. This could induce the production of inflammatory cytokines and activation of neutrophils and monocytes, accompanied by active free radical production, such as nitric oxide (NO), which will lead to gastric epithelial cell mutation and consequently result in inflammatory injury [29]. In the present study, ZZDX was found to significantly decrease the mRNA levels of IL-8 and NOD1 in gastric mucosa and downregulate the IL-1A, PGE2 and IL-6 levels in mouse serum, indicating that inflammation was significantly reversed [30]. Moreover, the PGE2 pathway plays a pivotal role in inflammation-induced gastric tumorigenesis [31], thus ZZDX might be able to inhibit the pathway of gastric tumorigenesis.

Subsequently, the mechanisms of reversing gastric mucosa inflammation were further explored using RNA sequencing and then verified in vivo and in vitro. According to the results of RNA sequencing, ZZDX could affect the pathways of unsaturated fatty acids biosynthesis, fatty acid elongation, and fatty acid metabolism. These pathways have been reported to be associated with ROS formation and can induce inflammation [32, 33]. The levels of ELOVL4, ACOT1 and SCD1 were significantly decreased after ZZDX treatment. These molecules are involved in the unsaturated fatty acid biosynthetic process [34–36]. ELOVL4, homologous to the ELO family which take part in fatty acid metabolism [37], has been reported in a gene metabolic signature, which is considered to be correlated with the overall survival (OS) and tumor immune microenvironment (TIME) in gastric cancer [38]. ACOT1, a gene for intracellular energy metabolism, could significantly promote the formation of gastric cancer tumor tissues and is associated with poor prognosis of gastric cancer [39]. SCD1, an enzymatic node which can convert saturated fatty acids into monounsaturated fatty acids, can promote the tumorigenesis of multiple cancers and has been considered to be a therapeutic target for some cancers [40]. In gastric cancer, SCD1 has been found to facilitate tumor growth and predict poor prognosis [41]. ZZDX treatment might rescue the progress of *H. pylori*-induced diseases by inhibiting these genes-associated molecular pathways in the pathogenic mechanisms.

However, we did not find a decrease in *H. pylori* infection status after treatment with ZZDX alone. Some previous studies have showed that ZZDX together with other medicines could be effective for *H. pylori* eradication. The eradication rate for *H. pylori* was found to be 77.8% [42] of ZZDX, together with Tibetan medicines such as Wuwei Shiliuwan, Ershiyiwei Hanshuishiwan, and Shiwuwei Heiyaowan. In addition, ZZDX with triple therapy of omeprazole, amoxicillin and clarithromycin could quickly improve clinical symptoms and effectively reduce the level of inflammatory indicators [11], and ZZDX with triple therapy of rebeprazole, amoxicillin and clarithromycin was more effective for the treatment of *H. pylori*-associated peptic ulcers in symptom relief rate and *H. pylori* eradication rate than those of conventional triple therapy [43]. Further studies should be performed to certify the potential clinical application of ZZDX together with other drugs in relieving symptoms and *H. pylori* eradication.

ZZDX is a complicated prescription composed of 35 mineral or animal or plant medicinal materials. Its chemical constituents are extremely complex, and it is really a tough work to identify its anti-inflammatory constituents. Activity guided chemical investigation is necessary to clarify the bioactive substance in the future researches. Here, the possible major bioactive constituents contributing to the preventive efficacy on *H. pylori*-induced inflammation of ZZDX are proposed through analysis of the prescription compositions and literatures. Calcite Lactis Praeparata, with calcium sulfate as the main constituent and accounting for about 15% of the total prescription amount of ZZDX, has been widely used for the treatment of gastric cancer and gastritis [44] and might be regarded as the main active component. Moreover, the characteristic anti-inflammatory constituents of cholanic acids (such as cholic acid, deoxycholic acid, ursodeoxycholic acid) [45, 46] and cycloketones (such as muscone) [47] in the animal medicines of Pulvis Fellis Ursi, Bovis Calculus (artificial) and Moschus (artificial), should play a key role in the rescue of *H. pylori*-induced inflammation. In addition, various anti-inflammatory constituents in the herbal medicines of ZZDX, such as sesquiterpene lactones in *Aucklandia lappa* Decne. [47] and *Inula racemose* Hook. f. [48], alkaloids in *Aconitum naviculare* (Bruhl.) Stapf [49], iridoid glycosides and phenylethanol glycosides in *Veronica eriogyne* H. Winkl. [50], organic acids in *Terminalia chebula* Retz. [51], and flavonoids in *Taraxacum officinale* F. H. Wigg [52]. and other herbs, might also be the important bioactive constituents that contributed to the gastric mucosa inflammation reversal.

It has been a field of current interest that diverse traditional medicines are evaluated for application against *H. pylori*. Many TCMs, such as turmeric, propolis, and garlic, have been reported to have anti-inflammatory, antioxidant, and antibacterial effects against *H. pylori* [53–56]. The anti-inflammatory and antioxidant effects of these medicines are mainly reflected in the inhibition of proinflammatory factors and reactive oxygen species generated by the interaction of *H. pylori* with gastric mucosa cells [15]. The antibacterial effects of TCMs against *H. pylori* have also been studied. On the one hand, these medicines can inhibit *H. pylori* enzymes such as urease, which decreases the acidity of gastric juice. On the other hand, they can inhibit the adhesion of *H. pylori* to gastric mucosa [57]. In addition, some TCMs that play an antibacterial role by targeting biofilms, proteins of the primary metabolism and virulence factors of *H. pylori*, have received more attention [58].

In view of that, it is suggested that TCM therapy cannot be used as monotherapy, although it has great potential to assist treatment [59, 60]. Furthermore, although there are many studies of TCM against *H. pylori* in vitro models, reliable randomized and controlled clinical trials which compare the efficacy of recommended triple therapies with herbal medicine on *H. pylori* treatment are still lacking [58]. Many factors must be taken into consideration, such as the identification, extraction and preparation of effective antibacterial components in herbal medicine, dose, formulation, dosing frequency, and duration of treatment. What needs to be illustrated is that some results of mechanistic exploration in this study showed obvious variation tendencies instead of significant changes, which might be due to the insufficient numbers of mice. This is a pilot study heralding the clinicopathological significance and mechanisms of ZZDX in *H. pylori* infection and provide clues for future studies.

## Data Availability

The authors hereby declare that the data and materials in this study will be presented upon request from the corresponding author.

## Abbreviations

ACOT1: Acyl-CoA thioesterase 1
A. D: Anno domini
BCA: Bicinchoninic acid
ELOVL4: ELOngation of Very Long-chain fatty acid-4
GO: Gene Ontology
H. pylori: Helicobacter pylori
HE: Hematoxylin–eosin
IHC: Immunohistochemical staining
MOIs: Multiplicities of infection
OS: Overall survival
PPARG: Peroxisome proliferator activated receptor gamma
PBS: Phosphate-buffered saline
RT-qPCR: Real-time quantitative PCR
ROS: Reactive oxygen species
SPF: Specific pathogen free
SCD1: Stearoyl-CoA desaturase 1
TIME: Tumor immune microenvironment
WHO: World health organization
ZZDX: Zuozhu-Daxi
